# Influence of Build Orientation, Geometry and Artificial Saliva Aging on the Mechanical Properties of 3D Printed Poly(ε-caprolactone)

**DOI:** 10.3390/ma14123335

**Published:** 2021-06-16

**Authors:** Ana C. Pinho, Ana P. Piedade

**Affiliations:** Centre for Mechanical Engineering, Materials and Processes (CEMMPRE), Department of Mechanical Engineering, University of Coimbra, 3030-788 Coimbra, Portugal; acdspinho@uc.pt

**Keywords:** 3D printing, geometry, build orientation, artificial saliva, mechanical properties

## Abstract

Additive manufacturing of polymers has evolved from rapid prototyping to the production of functional components/parts with applications in distinct areas, ranging from health to aeronautics. The possibility of producing complex customized geometries with less environmental impact is one of the critical factors that leveraged the exponential growth of this processing technology. Among the several processing parameters that influence the properties of the parts, the geometry (shape factor) is amid less reported. Considering the geometric complexity of the mouth, including the uniqueness of each teething, this study can contribute to a better understanding of the performance of polymeric devices used in the oral environment for preventive, restorative, and regenerative therapies. Thus, this work aims to evaluate 3D printed poly(ε-caprolactone) mechanical properties with different build orientations and geometries. Longitudinal and transversal toolpaths produced specimens with parallelepiped and tubular geometry. Moreover, as it is intended to develop devices for dentistry, the influence of artificial saliva on mechanical properties was determined. The research concluded that the best mechanical properties are obtained for parallelepiped geometry with a longitudinal impression and that aging in artificial saliva negatively influences all the mechanical properties evaluated in this study.

## 1. Introduction

Biomaterials for dental applications have been studied for many decades, leading to the development of new materials and have broadened their use in preventive, restorative, and regenerative treatments [1,2]. Metals and metallic alloys were the first class of materials with significant use in dentistry, such as in dental amalgams or, more recently, with titanium implants or nickel-titanium orthodontic brackets [3]. However, due to the increased longevity of the population and the rise of esthetics concerns, materials such as ceramics and polymers have gained their space in the dentistry field [4].

Particularly, the use of polymeric materials has found its applications to promote friction reduction [5], as drug delivery systems [6], antimicrobial activity [7], and corrective orthodontic devices [8]. Polymers that are used in dentistry applications vary from the most common such as polyethylene, poly(methyl methacrylate), and poly(ε-caprolactone) (PCL) to the less known as the case of polypyrrole and polyhexamethyldisilazane [9], to give some examples.

However, despite the evolution registered in the development of materials for dental applications, the processing techniques have registered very little progress. This trend can suffer a significant change with the advent of manufacturing techniques that conjugate personalization, geometry complexity, and reduction in raw material consumption and waste production, such as the case of additive manufacturing (AM). AM, commonly known as 3D-printing, is a group of processing techniques of metals, ceramics, and, particularly, polymers that have turned the corner from rapid prototyping to end-use production in the last decade parts and devices [10].

The properties of polymeric parts/components processed by 3D printing, including the mechanical ones, are highly dependent on the processing parameters. The most reported processing parameters that influence the final part properties include infill density, extrusion temperature, bed temperature, raster angle, and layer thickness [11,12,13,14]. However, the simultaneous study of the influence of geometry and build orientation is almost absent from the published literature. Moreover, for dental applications, the studies often evaluate the mechanical properties of the as-printed parts. They do not consider that the materials will be subjected to the oral wet environment, mainly constituted by saliva, during their lifetime. Additionally, it is known that both elastic moduli and the time-dependent viscoelastic response are important features as they modify the stress magnitudes sustained by the polymeric materials [15].

PCL is an aliphatic polyester that can be obtained by ring-opening polymerization of ε-caprolactone [16]. This semi-crystalline polymer has a unique set of properties such as slow biodegradability, solubility in a wide range of solvents, ease processability, hydrophobicity, and low melting temperature that stood out especially for biomedical applications [17,18]. Furthermore, by combining it with other polymers or even chemically modifying its polymeric chains, it is possible to fine tune the chemical and mechanical performance of PCL. Since its approval from the Food and Drug Administration of the United States of America (FDA) for biomedical applications, the research interest in PCL has increased exponentially [19]. In this field, PCL has been reported for the preparation of biomedical devices for regenerative medicine [19,20], orthopedics [21], drug delivery systems [22], and dental applications such as rings for implants [23], scaffolds for cell proliferation [24], implant coatings [25], or as functionalized prosthesis interfaces [26]. Considering the freedom that AM provides through the possibility of customization of medical devices, the possible applications can increase in all areas, including dentistry. These changes are beginning to be reported in cases where PCL reinforced with hydroxyapatite was 3D printed, resulting in scaffolds with good bioactivity according to the in vitro apatite-forming ability [27].

The main objective of the present work, and its contribution to the field, is to evaluate, simultaneously, the influence of the geometry and build orientation on the mechanical properties of 3D printed PCL parts obtained through tensile and stress relaxation tests. This is the first time that the geometry, also called shape factor, of 3D printed PCL parts, is addressed to the best of our knowledge. Due to envisaged application in the dentistry field, the same properties were evaluated after the printed specimens were aged in artificial saliva.

## 2. Materials and Methods

### 2.1. Materials

Poly(ε-caprolactone) (PCL) (Capa™6800; M_w_ = 80,000 g·mol^−1^) was gently offered by Perstorp (Warrington, UK) in granular form (approximately 3 mm pellets). The main properties of the PCL supplied by the manufacturer are summarized in Table 1.

### 2.2. 3D Printing

**Figure 1:**
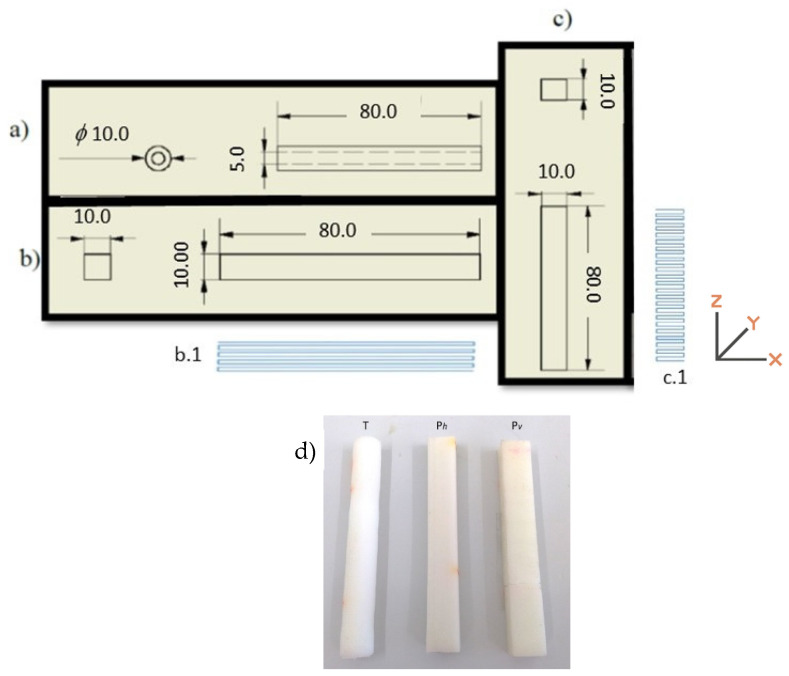
Schematic representation of the dimensions (in mm) of the printed specimens in the form of tube (**a**) and parallelepiped (**b**,**c**) with the build orientation for the two different parallelepiped forms (**b.1** and **c.1**); (**d**) digital macrographs of the three printed specimens.

The PCL test pieces were 3D printed using a Bioextruder (homemade) equipment, equipped with a mini extruder (homemade) that allows the use of the polymer in the form of granules. The test pieces were printed with two distinct geometries, in the form of a tube, printed with a horizontal build orientation (T) and parallelepiped (P). The latest was printed horizontally (longitudinal, X-Y) (P_h_) and vertically (transverse, X-Z) (P_v_), as schematically shown in Figure 1, where the dimensions of each specimen are also presented. The same figure also presents the digital macrographs of the three printed specimens.

For all the samples, the main printing parameters were kept constant: nozzle diameter of 0.4 mm, nozzle temperature at 80 °C, spindle rotation speed of 22 rpm, and printing speed of 25 mm·min^−1^.

### 2.3. Swelling Capacity

The swelling capacity of the printed specimens was determined in artificial saliva (AS) with pH 7.4, prepared according to previously reported chemical composition, AFNOR (Association Française de Normalization) [28]. Before the tests, the printed specimens were dried for 24 h at 50 °C and weighted. They were immersed in AS for 120 h, at 37 °C, and placed in the thermos shaker (C. Gerhardt Analytical Systems, Königswinte, Germany) at 100 rpm. At the end of the tests, the excess of AS was gently removed using filter paper. The swollen specimens were weighted, and the swelling capacity calculated using Equation (1):

(1) $$\mathrm{Swelling}\;\mathrm{Capacity}\;\left(\%\right)=\frac{W_{s}-W_{d}}{W_{d}}\times 100$$

where *W_s_* is the weight of the swollen samples while *W_d_* refers to the weight of the dried specimens. All measurements were performed in triplicate.

### 2.4. In Vitro Test with Artificial Saliva

The printed specimens were placed in 15 cm^3^ Falcon tubes (Orange Scientific, Braine-l’Alleud, Belgium) filled with artificial saliva AFNOR [28]. Each set was placed inside a thermos shaker (C. Gerhardt Analytical Systems, Königswinte, Germany) at 37 °C and 100 rpm for 15 days. After the incubation period, the samples were cleaned for the excess liquid and immediately tested for determining the mechanical properties.

### 2.5. Tensile Tests

For the printed specimens, as-printed and after aging in AS, tensile tests were performed in an Autograph AGS-X, from Shimadzu (Kyoto, Japan), with 100 kN maximum load, an MFA25/12 mechanical extensometer (Kyoto, Japan), and the software Trapezium–X according (Kyoto, Japan). A gauge length of 50 mm and a displacement rate of 3 mm·min^−1^ were used in these measurements. In the specimens printed with a tube shape, a metallic rod was placed inside the tube in the part that was hold by the grips to avoid the collapse of the tube. Three valid tests for each specimen were considered to estimate the mean average value and respective standard deviation.

### 2.6. Stress Relaxation Tests

The stress relaxation test consists of applying a constant tension in the elastic region to induce an initial strain (ε_0_) and, over time, registering the decrease in the applied tension. The tests were performed in the same equipment and under the same test conditions as the tensile tests. The total duration of the tests was 10,800 s.

The stress relaxation time value (τ) is characteristic of each polymeric material at a specific temperature. According to the Kohlrausch–Williams–Watts relaxation model [29], τ is calculated according to Equation (2):

σ = σ_0_ exp (−t/τ) (2)

where σ_0_ is the value of the initially applied stress and σ the value of the stress after time t. For each specimen, three valid tests were obtained to estimate the mean average and standard deviation values. Additionally, the relaxation modulus (E_R_) can be calculated [30] according to Equation (3):

E_R_ = σ_(t)_/ε_0_ (3)

where σ_(t)_ is the tension after time t, and ε_0_ is the initial strain due to the applied stress.

## 3. Results and Discussion

### 3.1. Swelling of Artificial Saliva

The results from the swelling percentage of the 3D printed specimens in artificial saliva are presented in Table 2. The results were also normalized considering the area exposed to the saliva due to the different geometries of the parts.

The AS swelling for all the tested geometries was low, as expected, considering that PCL, with an average molecular weight of 80,000 g·mol^−1^, is considered a hydrophobic polymer [31]. Nonetheless, as the polymer used for printing the three types of specimens is the same, it would be expected that, for similar areas exposed to the fluid, the percentage of AS sorption would be identical. The values of the normalized sorption per unit area show that the specimens T and P_v_ have similar values. However, P_h_ specimens have lower fluid sorption values. Since the polymer is the same, these values raise the suspicion that the adhesion between successive layers is not similar in the three types of test pieces. Although T and P_h_ were printed with the same build orientation, the geometry of the specimen seems to influence the interlayer adhesion. This assumption will reflect a greater free volume between layers and, consequently, a greater area available for the interaction with the artificial fluid.

### 3.2. Mechanical Properties

#### 3.2.1. As-Printed

**Figure 2:**
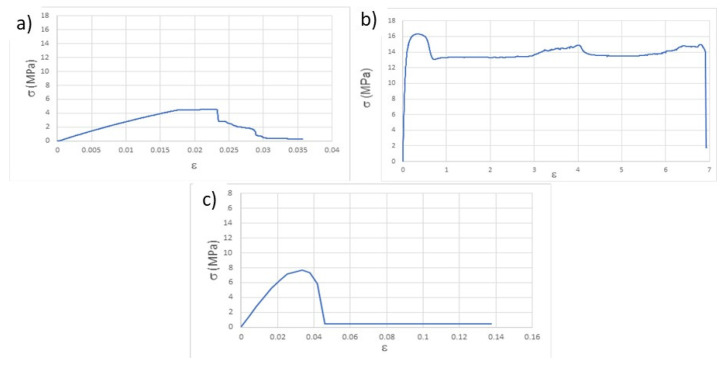
Representative stress-strain curves resulting from the tensile tests of the as-printed specimens: (**a**) T, (**b**) P_h_, (**c**) P_v_.

The determination of the mechanical properties through the tensile test was carried out, first, on the specimens after printing. From the stress-strain plots (σ vs. ε), as exemplified in Figure 2 and according to the ASTM D638 (American Society for Testing and Materials, D638-Standard Test Method for Tensile Properties of Plastics) standard, the following values were determined: Young’s modulus, for 0.02 deformation (E), elastic limit stress, also for deformation of 0.02 (σ), stress at break (σ_b_) and deformation at break (ε_b_). These last two values were assumed to be those immediately before the abrupt breakdown of the stress values because, due to the viscoelastic behavior of the thermoplastic used in the study, sometimes the fracture of the specimens was not attained. The summary of the calculated values is presented in Table 3.

**Figure 3:**
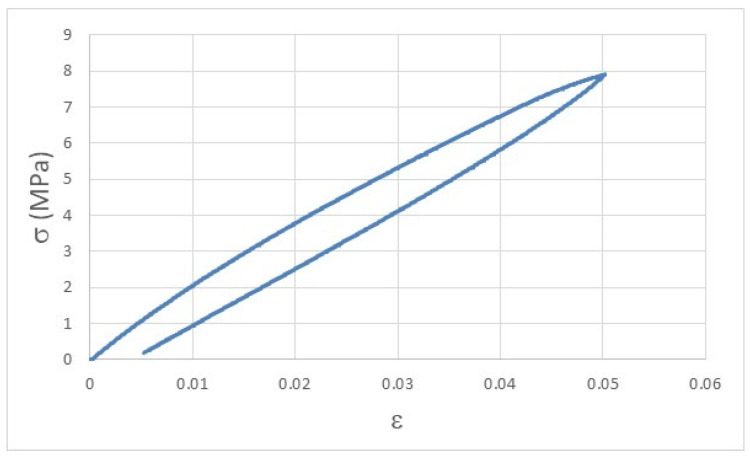
Stress-strain curve of a P_h_ printed specimen after 1 cycle of load-unload experiment, highlighting the permanent deformation due to the elastic behavior of polymeric materials.

The viscoelastic behavior of polymeric materials can be easily understood by the curve displayed in Figure 3, where, after the removal of the load that imposed an elastic deformation, the P_h_ printed sample did not fully recover the initial dimensions (ε = 0) and presents a permanent deformation of 0.005 after just one cyclic deformation.

This behavior is responsible for the stress-relaxation of polymers, and it is very important to be evaluated as it establishes the deformation over time that polymers suffer during their life cycle.

**Figure 4:**
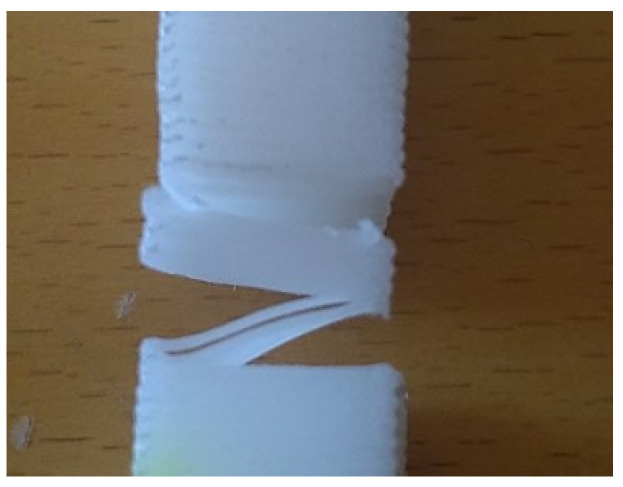
Macrographic image showing the loss of cohesion between the printed layers of the P_v_ specimen.

**Figure 5:**
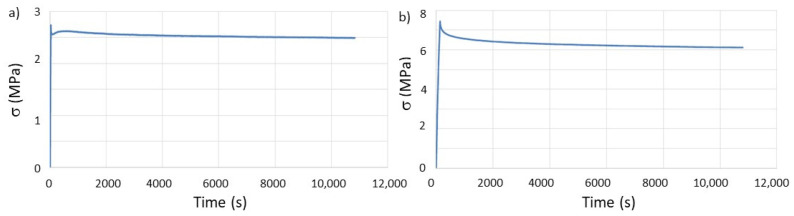
Evaluation of stress relaxation behavior, over time, of the as-printed specimens: (**a**) T and (**b**) P_h_.

From the previous tensile test results, it was clear that the initial load to be applied to each of the printed specimens for the stress-relaxation evaluation could not be the same, as the elastic deformation domain is different for each geometry and building orientation. Therefore, preliminary tests were conducted to evaluate the appropriate tension to apply to each type of printed specimen. From these tests, it was immediately clear that the P_v_ geometry and building orientation did not satisfy the requirements in use, as the layers lost their interconnectivity (Figure 4). Consequently, only the T and P_h_ specimens were tested with initial tensions of 2.75 and 7.5 MPa, respectively. The graphics from the stress-relaxation tests are presented in Figure 5, and the calculated values are summarized in Table 4.

The most significant result for this set of tests is the variation of τ. When tested in the same environmental conditions of temperature and relative humidity, the relaxation time for the same polymer is known to be a constant value [32]. However, this work demonstrates that 3D printed polymers, due to the well-documented [14,33,34] anisotropic properties induced by several printing parameters, alters the previously established knowledge for bulk polymers. Moreover, considering that the building orientation is the same for the T and P_h_ specimens, these results show that the geometry of the printed component/part greatly influences the mechanical properties. This observation can be helpful when designing for AM with the purpose of shape-morphing with time, i.e., 4D printing [35,36], and taking advantage of the different relaxation behavior of the same material depending on the chosen geometry.

Considering that most often studies for evaluating mechanical properties 3D print their specimens according to ASTM or ISO standards and that these geometries are not the ones that are going to be used for the manufacturing of the final part, the reported results in this work raise concerns regarding the real value of the properties of the final part.

#### 3.2.2. After In Vitro Aging

**Figure 6:**
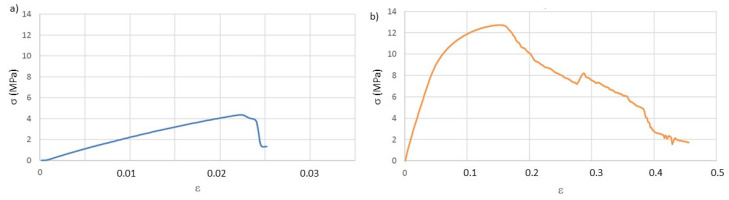
Representative stress-strain curves after aging in AS (artificial saliva): (**a**) T and (**b**) P_h_.

The tensile tests were done only in the T and P_h_ specimens, as the P_v_ showed very poor results, even in dry conditions, in the stress relaxation tests. Representative stress-strain curves are presented in Figure 6, and the obtained results are in Table 5.

As expected, the mechanical properties suffered a decrease in their values after aging in AS. The results indicate that the water-based solution acts as a plasticizer. This action is mainly observed in the specimens printed with T geometry, which are the ones that presented higher normalized AS swelling when compared with the P_h_ geometry. The T geometries suffered a decrease of the Young modulus and strain yield of around 33%, while the P_h_ geometry presents, for the same properties, a decrease of 15%.

**Figure 7:**
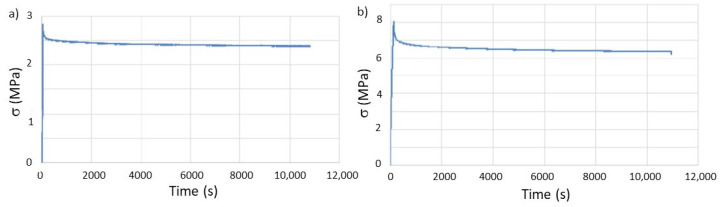
Evaluation of stress relaxation behavior, over time, of the printed specimens after in vitro aging: (**a**) T and (**b**) P_h_.

The stress relaxation tests were performed with the same parameters as those used on the as-printed geometries, as shown in Figure 7. The results from the tests are summarized in Table 6.

The results show that, although the relaxation module does not present significant changes when compared to the specimens tested after printing, the relaxation time drastically decreases in both types of geometries, with particular emphasis on the tube test pieces, where the value is half the one previously calculated. It must be emphasized that this geometry was the one with the highest percentage of AS sorption due to more free volume between layers.

Consequently, the obtained result is expected when considering that the polymer will deform to cancel the stress to which it is subjected during stress relaxation. If a plasticizer is added to the polymer, in this study the AS, the interaction between adjacent macromolecules becomes weaker due to reducing the number of secondary bonds. Consequently, the deformation of the polymer, which can be regarded as the slipping inter-macromolecules, is facilitated. This process is thermodynamically more favorable when the percentage of molecules with plasticizing effect is higher. Effectively, the amount of AS absorbed by the PCL with a T geometry is higher than for P_h_.

## 4. Conclusions

AM processing techniques applied to the health sector, particularly in dentistry, have experienced an exponential increase in the last years. According to the envisaged application, the dependence of the properties on the printing parameters obliges detailed studies. There is an established number of processing parameters that impact the mechanical properties of the printed specimens. However, geometry is not one of the reported factors when optimizing the processing process by material extrusion. This fact raises some questions considering the mechanical properties evaluated in printed specimens with geometries imposed by ASTM or ISO standards significantly different from the real devices/components/parts for a specific application.

In this work, we have demonstrated that geometry critically influences mechanical properties. Consequently, this work shows the need to adapt the processing parameters, performed tests, and characterization techniques to the desired final application. Moreover, it is urgent to develop and implement specific standards for 3D printing, as the current ones were intended to be applied to bulk polymers and as shown in the current work, are not adjusted to the new challenges raised by 3D printing.

Additionally, the real performance of 3D printed components must be evaluated or studied using environments that mimic the natural milieu where the design device will operate and not only in the as-processed devices/specimens. As demonstrated in the current work, artificial saliva greatly influences the mechanical properties and can even jeopardize the use of a particular polymer, in this case PCL, in the dentistry field. Considering the obtained results, a study involving in vitro tests with prokaryotic cells and geometries with real use in dentistry, such as retainers or temporary scaffolds, will be one of the routes to explore as future perspectives.

## Figures and Tables

**Table 1:** Properties of PCL, according to the supplier.

Property (Units)	Value
Melt flow index (g/10 min)	4.03–2.01 ^1^
Water content (%)	0.35 (max)
Average ponderal molecular weight (g/mol)	80,000
Fusion temperature (°C)	56–60
Glass transition temperature (°C)	−60
Strain at break (%)	800
Young modulus (MPa)	400
Tensile strength (MPa)	16

^1^ Load 5 Kg, nozzle 2.5 cm, temperature 160 °C.

**Table 2:** Swelling of artificial saliva of the 3D printed PCL specimens.

Printed Specimens	AS Swelling (%)	Normalized AS Swelling (%·mm^−2^)
T	5.1 ± 0.4	1.3 × 10^−3^
P_h_	0.8 ± 0.1	1.9 × 10^−4^
P_v_	4.6 ± 2.1	1.1 × 10^−3^

**Table 3:** Mean and standard deviation values of the mechanical properties calculated from the tensile tests of the as-printed PCL specimens.

Specimen	E_0.02_ (MPa)	σ_0.02_ (MPa)	σ_b_ (MPa)	ε_b_
T	105 ± 13	2.1 ± 0.6	0.5 ± 0.1	0.035 ± 0.0
P_h_	735 ± 15	14.7 ± 2.4	14.1 ± 0.9	6.9 ± 0.3
P_v_	310 ± 20	6.2 ± 1.7	0.5 ± 0.1	0.05 ± 0.0

**Table 4:** Mean and standard deviation values of the mechanical properties calculated from the stress relaxation tests of the as-printed PCL specimens.

Specimen	E_R_(1) (MPa)	τ (h)	Δσ (%)
T	200 ± 10	13 ± 0.3	9.5 ± 0.5
P_h_	126 ± 8	6.8 ± 0.1	17.3 ± 1.0

**Table 5:** Mean and standard deviation values of the mechanical properties calculated from the tensile tests of the PCL (poly(ε-caprolactone)) specimens after aging in AS.

Specimen	E_0.02_ (MPa)	σ_0.02_ (MPa)	σ_b_ (MPa)	ε_b_
T	70 ± 7	1.4 ± 0.1	1.3 ± 0.1	0.025 ± 0.0
P_h_	630 ± 8	12.6 ± 1.3	1.7 ± 0.2	0.46 ± 0.1

**Table 6:** Mean and standard deviation values of the mechanical properties calculated from the stress relaxation tests of the printed specimens after in vitro aging.

Specimen	E_R_(1) (MPa)	τ (h)	Δσ (%)
T	192 ± 9	6.5 ± 0.5	12.5 ± 1.7
P_h_	130 ± 9	4.8 ± 0.3	19.4 ± 1.2

## Data Availability

All the data is available within the manuscript.
